# Prevention, Cessation, or harm reduction: Heterogeneous effects of an intimate partner violence prevention program in eastern Democratic Republic of the Congo

**DOI:** 10.1371/journal.pone.0282339

**Published:** 2023-03-08

**Authors:** Alev Gurbuz Cuneo, Julia Vaillant, Estelle Koussoubé, Rachael S. Pierotti, Kathryn Falb, Rocky Kabeya

**Affiliations:** 1 Africa Region Gender Innovation Lab, Office of the Chief Economist, The World Bank Group, Washington, DC, United States of America; 2 International Rescue Committee, Washington, DC, United States of America; 3 International Rescue Committee, Kampala, Uganda; National Institute of Allergy and Infectious Diseases, UNITED STATES

## Abstract

**Introduction:**

The Engaging Men through Accountable Practice (EMAP) program is a series of facilitated group discussions for men in the Democratic Republic of the Congo that sought to reduce intimate-partner violence and transform gender relations. While a previous analysis found null impacts on women’s experience of past-year intimate-partner violence (IPV), these average results obscure important heterogeneity. The study objective is to analyze the effects of EMAP on subgroups of couples based on their initial levels of IPV.

**Methods:**

We use two rounds of data (baseline and endline) collected from adult men (n = 1387) and their female partners (n = 1220) as part of a two-armed, matched-pair, cluster randomized controlled trial conducted between 2016 and 2018 in eastern Democratic Republic of the Congo. Loss to follow up was low as 97% of male and 96% of female baseline respondents were retained at endline. We define subgroups of couples based on their baseline reports of physical and sexual IPV using two different methods: i) subgroups determined by binary indicators of violence at baseline, and ii) Latent Class Analysis (LCA).

**Results:**

We find that the EMAP program led to a statistically significant decrease both in the probability and severity of physical IPV among women who experienced high physical and moderate sexual violence at baseline. We also find a decrease in the severity of physical IPV (significant at the 10% level) among women who experienced both high physical and high sexual IPV at baseline. Findings indicate that the EMAP program was more effective at reducing IPV perpetration among men who were the most physically violent at baseline.

**Conclusion:**

These results suggest that men who perpetrate violence against their female partners with greater severity than average may be inspired to reduce their use of violence through participatory discussion with less violent men. In contexts of endemic violence, programs like EMAP can lead to a meaningful short-term reduction in harm to women, perhaps even without transforming prevailing norms about male superiority or the acceptability of IPV.

**Trial registration:**

**Trial registration number:** NCT02765139.

## Introduction

Programming that engages men and boys is now a common component of efforts to prevent violence against women and to promote gender equality [1, 2]. A growing body of research has shown that these types of interventions hold promise, especially for the reduction of intimate-partner violence (IPV), although programs vary substantially both in their design and effectiveness [3]. In addition to differences across programs, more recent studies have suggested that individual programs have heterogeneous impacts on IPV prevention. Gibbs et al. [4] re-analyzed data from three impact evaluations of prevention programs and find that each intervention had meaningful positive impact on about 20 percent of participants. The analysis showed that men with different baseline characteristics related to mental health, poverty, and attitudes about violence responded differently to the prevention interventions. These findings highlight the need for more research on which participants benefit from prevention programs to expand our understanding of the mechanisms of change, and to improve program design.

In a recent paper, Vaillant et al. [5] estimated the average treatment effects of the Engaging Men through Accountable Practice (EMAP) program in the Democratic Republic of the Congo (DRC). The study found significant reductions in men’s intention to commit violence, increases in gender equitable attitudes and behaviors, as well as improvement in relationship quality as reported by women, but no reductions in women’s reports of past year IPV. EMAP is a men’s discussion group program that sought to transform gender relations by creating a cadre of male allies who practice and promote gender equity and do not perpetrate IPV or other forms of violence. The program was designed for men who are not violent toward women and girls. As such, EMAP differed substantially from batterer intervention programs, which have been shown to be mostly ineffective [6, 7]. Nonetheless, it is neither feasible nor ethical to screen potential participants based on their history of perpetration of IPV. The EMAP baseline survey documented substantial variation in the rates of previous year physical and/or sexual violence reported by female partners of men who had volunteered to participate in the program. This reality begs the question: although there was no significant impact on average, did program impact vary across men who were relatively more or less violent at baseline?

This paper tests for the differential impacts of EMAP depending on baseline types and levels of violence. Building on recent work (e.g., [4]), the analysis additionally investigates binary and severity measures of violence to capture cessation, primary prevention, and harm reduction. The few existing studies that examine heterogeneous impacts of IPV prevention programs find inconsistent results. Chatterji et al. [8] use data from the impact evaluations of the Stepping-Stones/Creating Futures (SS-CF) intervention in South Africa, and the Indashyikirwa intervention in Rwanda. The subgroup analyses show that the Indashyikirwa intervention was more effective at reducing or stopping ongoing (women’s) experience and (men’s) perpetration of IPV than at preventing its onset. In contrast, the effects of the SS-CF intervention reported by Gibbs, et al. [4] seem to be mainly driven by the impacts on male participants who did not report perpetration of IPV at baseline. Chatterji et al. [8] attribute the differences in results between the two programs to differences in the intervention participants (older men mainly in couples in Indashyikirwa versus younger unmarried men in SS-CF) and differences in the content of the interventions. Relatedly, Christofides et al. [9] use a cluster randomized controlled trial to evaluate the effectiveness of the Sonke CHANGE intervention, which targeted young men in a peri-urban community in South Africa. While acknowledging limitations due to study design, Christofides et al. [9] indicate that the analysis suggests a greater reduction of IPV among men who were least violent at baseline.

It is theoretically ambiguous whether IPV prevention programs have the potential to achieve greater positive impact on the behaviors of relatively less or more violent men. There are reasons to believe that the least violent men will be most substantially affected by IPV prevention programs. First, less violent men may be more open to change. Lower levels of violence perpetration are generally associated with a less favorable attitude toward IPV [10]. Second, research on social norms change interventions for the prevention of other types of gender-based violence (GBV) finds that the impact is greatest among individuals whose pre-intervention attitudes were more aligned with the targeted change (e.g., [11] regarding female genital cutting). Together, these studies suggest that men who are more rejecting of IPV and who are less violent will be more open to the changes encouraged by IPV prevention programs. Furthermore, prevention of the onset of use of violence (called primary prevention) among men who are already non-violent may be relatively easier to encourage as it aligns with the well-documented behavioral and psychological preference for inertia [12, 13]. Finally, research consistently suggests that for men there is a strong association between the perpetration of severe forms of violence and poor mental health, substance abuse, childhood trauma, and other antisocial behaviors [4]. Recent transdiagnostic cognitive behavior therapy interventions have demonstrated that treating all these problems concurrently can lead to reductions in men’s use of IPV [14, 15]. It is plausible, therefore, that to achieve impact among men perpetrating high levels of violence in their households there is a need for specialized mental health treatment rather than more general behavior change programs.

On the other hand, in-depth knowledge of the context, program participants, and intervention design support a hypothesis for why relatively more violent men would be encouraged to change by participation in EMAP. The program may cause more violent men to learn that their use of violence is outside norms of accepted behavior. In-depth qualitative research in the study communities prior to and during the intervention found that IPV is tolerated as a means of disciplining wives, but it is not encouraged [16]. Moreover, in the population targeted for program participation, men are celebrated for achieving “harmony” in their homes, and violence is seen to undermine harmony, indicating support for a non-violent masculinity. Also, like the Indashyikirwa intervention in Rwanda, EMAP participants are, on average, middle aged and married. EMAP did not specifically target economically disadvantaged men. Thus, the target population and the normative context contrast with other settings—including those where SS-CF and the SONKE Change intervention were implemented—where pressure to perform a youthful violent hypermasculinity, and the inaccessibility of a traditional masculinity based on the provision of economic support to women, influence men’s behaviors [17, 18]. Finally, EMAP is a group-based program where participants are encouraged to engage in discussion and thereby learn from each other’s reports of their behaviors and preferences regarding IPV. With all of this in mind, it is reasonable to hypothesize that men who are using relatively high levels of IPV at baseline will be encouraged to reduce their use of violence.

To examine these hypotheses, this paper presents heterogeneity analysis of the effects of EMAP on groups of couples defined using two different methods: i) subgroups based on binary indicators of initial levels of violence, ii) and Latent Class Analysis (LCA). LCA builds subgroups that allow for a combination of different types of violence and better reflects the complexity of IPV. While Vaillant et al. [5] only measured experience of past-year IPV using binary indicators, in this paper we use continuous measures of IPV in addition to binary indicators of violence to examine not only prevention and cessation, but also harm reduction [4].

## Methods

### Study design

We use two rounds of survey data collected in 28 communities (hereafter, sites) in North and South Kivu provinces, the DRC, as part of a matched-pair, cluster randomized controlled trial conducted between 2016–2018. 30 sites, originally part of the research, were selected in coordination with the International Rescue Committee (IRC), the implementing organization, based on their implementation capacity. The sites were then matched based on sociodemographic characteristics, so that within each pair of sites, one site was randomized to either treatment or control arms. Out of these 30 sites, baseline data collection could only be conducted in 28 sites (14 control sites and 14 treatment sites) due to security concerns in the region. More details on the randomized controlled trial can be found in Vaillant et al. [5]. The randomized controlled trial was complemented by in-depth longitudinal qualitative data collection throughout program implementation [16].

### Intervention

EMAP is a series of facilitated group discussions for men that sought to transform gender relations in communities by creating a cadre of male allies who practice and promote gender equity and do not use violence. The program was designed to give male participants the tools and knowledge to rethink belief systems and prevent GBV through individual behavioral change. EMAP invites the same group of men to participate in 16 weekly discussions with their male peers. The sessions are led by male trained facilitators. Topics explored the underpinnings of masculinity; types, causes and consequences of violence against women and girls; and opportunities for positive role modeling and reflection on their own power and privilege. Women’s groups were established prior to launching male discussion groups to promote accountability to the needs, views, and priorities of women in the community. Discussion topics that arose in the women’s groups were communicated to the men’s groups throughout the intervention and their reflections were incorporated into the facilitators’ approach. Men in the control sites participated in alternative group training sessions on topics such as poultry rearing, woodworking, tree planting, or driving lessons.

### Target population

Adult men, aged 18 years and older, were eligible to participate in the study. Additional inclusion criteria included having lived in the community for at least six months with plans to continue living there for at least an additional six months, ability to actively participate in the group, non-involvement with an ongoing evaluation of adolescent girl programming that was operational in some sites, and committing to not perpetrate violence for the duration of the intervention. Female partners of men were also interviewed if they were above 15 years of age.

### Data collection

Baseline data collection occurred between April and September 2016 and a follow-up survey was conducted between September and December 2017. Men and their female partners were invited to participate in the interviews. Electronic data collection was completed by gender-matched enumerators. For sensitive outcomes, audio computer assisted self-interviews (ACASI) were used in order to limit potential under-reporting. The survey was developed in French and subsequently translated and back translated into Kiswahili, Mashi, and Kinyarwanda languages. Ethical approval was received from International Rescue Committee’s Internal Review Board with approval number IRB #: 00009752 on March 7, 2016 and from the DRC Ministry of Women, Family, and Children with approval number # 433/DR/IRC/015. Informed oral consent was obtained from all participants and established global guidelines on conducting ethical violence against women research were followed. Waiver of parental consent was obtained for participants ages 15–17.

At baseline, 1,387 men and 1,220 women were interviewed. Loss to follow up was low as 97% of male and 96% of female baseline respondents were retained at endline. S1 Fig shows the trial profile. The most common reason for attrition was inability to locate the respondent, followed by refusal and having moved to a different location.

### Measures

We define four primary outcomes indicators, all reported by the female partner of male participants: (1) binary past-year physical IPV, (2) past-year severity of physical IPV, (3) binary past-year sexual IPV, (4) past-year severity of sexual IPV. Table 1 describes the construction of the outcomes of interest.

**Table 1:** Description of the outcomes.

Indicator	Construction
Physical IPV (binary)	Binary variable coded to 1 if a female partner of a male participant responded affirmatively to an instance of any of the following items occurring in the past 12 months: (1) partner pushed, shook or threw something at respondent, (2) slapped her, (3) twisted respondent’s arm or pulled her hair, (4) punched respondent with his fist or with something that could hurt her, (5) kicked respondent, dragged her on the floor, beat her, (6) tried to choke respondent or burn her on purpose, (7) threatened or attacked respondent with a knife, gun or other weapon. Coded to 0 if no occurrence, coded to missing if one item missing and all others are 0, coded to 1 if one item missing and at least one item is 1.
Physical IPV Severity Index (continuous)	Continuous variable that is the standardized average of the responses to the 7 physical violence items listed above that report the frequency of instances of each type of physical violence reported in the past 12 months on a 4-point Likert frequency scale (0 = Never, 1 = Once, 2 = Sometimes, 3 = Often). Coded to missing only if all the items are missing. Range is 0–3.
Sexual IPV (binary)	Binary variable coded to 1 if a female partner of a male participant responded affirmatively to an instance of any of the following items occurring in the past 12 months: (1) partner physically forced respondent to have sex with him even when she did not want to, (2) forced respondent to have sex even when she did not want to because she was afraid of what he could do to her, (3) forced respondent to do sexual acts respondent finds humiliating. Treatment of missing values as above.
Sexual IPV Severity Index (continuous)	Continuous variable that is the standardized average of the responses to the 3 sexual violence items listed above that report the frequency of instances of each type of sexual violence reported in the past 12 months on a 4-point Likert frequency scale (0 = Never, 1 = Once, 2 = Sometimes, 3 = Often). Coded to missing only if all the items are missing. Range is 0–3.

Alternative constructions of the IPV severity indices that handle item missingness differently are considered as robustness checks. Missing responses to questions about IPV experience might not be random. In response, we considered three versions for each IPV severity variable: (a) index is coded to missing if any item was missing, (b) missing items are replaced with “0 = Never” if there is at least one non-missing item, (c) missing items are replaced with the mean of the item in the control group if there is at least one non-missing item in her responses.

### Statistical analysis

To examine the differential impacts of EMAP by baseline experience of IPV reported by women, subgroups of couples are constructed using two different methods. First, subgroups of couples are defined using the binary indicators of IPV experience in the last 12 months, for physical and sexual violence. Throughout the paper, we use the terms “physical and sexual IPV” and “physical and sexual violence” interchangeably. A couple is in the “physically violent” subgroup if the female partner of the male participant reported an instance of physical violence perpetrated by her intimate partner in the 12 months prior to baseline. Similarly, a couple is in the “physically non-violent” subgroup if the female partner reported at baseline that none of the instances of physical violence had occurred in the last 12 months. The same methodology is followed to form the subgroups of couples for sexual violence. The sexually and physically violent subgroups at baseline are not mutually exclusive.

Second, to explore distinct patterns of IPV experience, a Latent Class Analysis (LCA) was conducted to identify latent subgroups (i.e., latent classes) of couples exposed to physical and sexual violence at baseline. LCA is a method for identifying and understanding unobserved groups in a population [19]. In LCA models, classes are identified in a way where each class is distinct from one another and the individuals within classes have similar responses to the items included in the analysis [20]. Unlike the subgroups based on binary indicators, LCA considers co-occurrence of different types of violence. Researchers are increasingly using LCA to identify subgroups of IPV exposure. Previous work relying on LCA to identify distinct classes of violence have explored the association of these classes with mental health outcomes [20–23], paid work disruptions due to IPV [24], and perpetration of IPV [9].

Physical and sexual IPV experience as reported by female partners are measured with 7 physical violence items and 3 sexual violence items (Table 1). We used these 10 IPV items to fit a series of latent class models with various number of classes (from 2 to 4). The baseline frequency of each item used is reported in S1 Table 1 in the S1 Appendix. The best-fitting model is detected by selecting the model with the largest log-likelihood, and the smallest values of the Akaike Information Criterion (AIC) and Bayesian Information Criterion (BIC) and examining the entropy measure (See S1 Table 2 in the S1 Appendix).

Having defined these subgroups of couples based on the female partner’s experience of IPV at baseline, we conduct an intention-to-treat analysis of the impacts of EMAP separately for each subgroup of couples identified through both methods using ordinary least squares (OLS) and linear probability (for binary outcomes) models. Our findings are robust to using logistic models. Results are available upon request. We estimate adjusted models in which we control for the following baseline characteristics: men and women’s age and education, household size, and the language of the interview. For all analyses, standard errors are clustered at the site level in the regressions.

## Results

### Descriptive statistics

The sample is well balanced across control and treatment arms in baseline characteristics (Table 2). On average, women were around 35.2 years old in the control group and 35.9 years old in the EMAP group. The average age of men was 40.6 years old and 41.2 in the control and EMAP groups respectively. This is notably older than the average age of men in similar studies [4, 8, 9]. In addition, 36% of women in the control group and 37% of women in the treatment group report having experienced physical IPV in the past year at baseline, while 32% of women at baseline reported having experienced sexual IPV.

**Table 2:** Baseline characteristics by study arm.

		(1)		(2)	
		**Control group**		**Treatment group**	**p-value**
	**N**	**Mean[SD]**	**N**	**Mean[SD]**	**(1)-(2)**
*Control variables*					
Total household size (number)	639	7.272	645	7.253	0.952
		[5.820]		[5.956]	
Woman respondent’s age (years)	585	35.234	608	35.944	0.639
		[24.806]		[27.833]	
Woman respondent partner’s age (years)	704	40.624	704	41.151	0.717
		[28.047]		[26.841]	
Woman has primary education (binary)	608	0.273	621	0.258	0.698
		[0.774]		[0.615]	
Woman has secondary or higher education (binary)	608	0.197	621	0.169	0.644
		[0.951]		[1.198]	
Woman’s partner has primary education (binary)	602	0.279	617	0.305	0.624
		[0.960]		[0.873]	
Woman’s partner has secondary or higher education (binary)	602	0.505	617	0.451	0.574
		[1.586]		[1.807]	
*IPV Experience reported by women*					
Physical IPV (binary)	569	0.362	572	0.371	0.887
		[1.231]		[0.787]	
Sexual IPV (binary)	582	0.344	589	0.299	0.200
		[0.757]		[0.364]	
Physical IPV Severity Index	602	0.230	617	0.215	0.794
		[1.269]		[0.647]	
Sexual IPV Severity Index	603	0.342	609	0.276	0.250
		[1.271]		[0.576]	

Note: The last column displays p-values from t-tests of differences in the means between the control group and the treatment group. Standard deviations are clustered at site level. *Indicates significance at 10% level, ** at 5% level, *** at 1% level. N denotes the number of observations. The number of observations varies slightly across variables due to missing observations.

**Fig 1:**
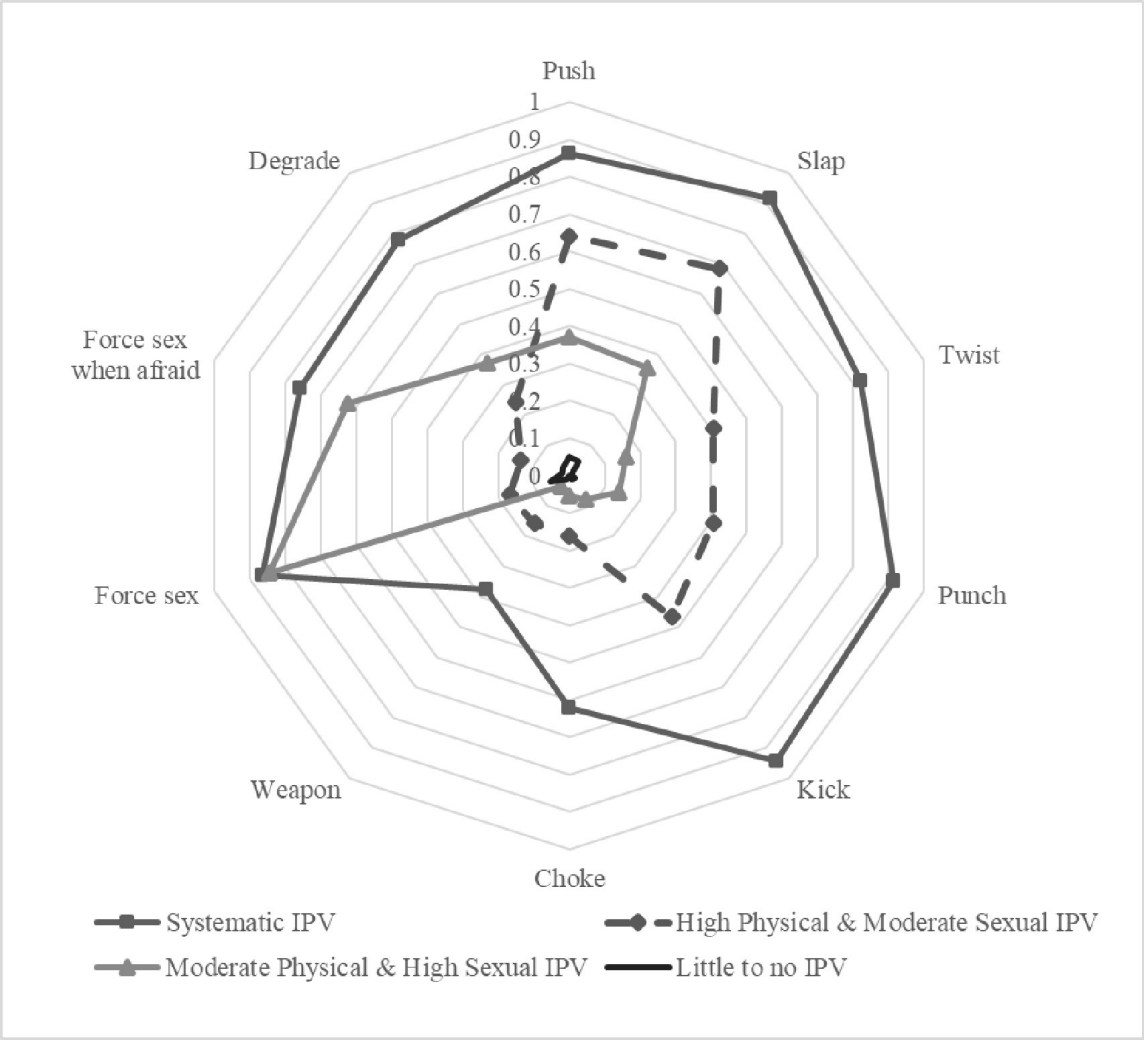
Conditional item probability plot.

Fig 1 presents the conditional item probabilities of each IPV item for each distinct class identified using LCA. We named the identified classes according to their unique characteristics based on the probabilities of the indicators in each class. Table 3 presents the distribution of our sample across the latent subgroups by experimental arm. They are: (i) Little to no IPV at baseline, (ii) Moderate Physical & High Sexual IPV at baseline, (iii) High Physical & Moderate Sexual IPV at baseline, (iv) Systematic IPV at baseline, where the woman reported high levels of both physical and sexual violence at baseline.

**Table 3:** Latent subgroups of couples defined using women’s experience of IPV at baseline.

Classes	Control group (N)	Treatment group (N)	Total (N)	Total (%)
Systematic IPV	40	32	72	5.9
High Physical & Moderate Sexual IPV	46	61	107	8.77
Moderate Physical & High Sexual IPV	102	85	187	15.33
Little to no IPV	415	439	854	70
Total	603	617	1,220	100

Notes: N denotes the number of observations.

More than two thirds of women (70%) in our sample reported little to no experience of IPV in the past 12 months. 15.3% of the sample experienced moderate physical violence and high levels of sexual IPV, while 8.8% of our sample was exposed to high levels of physical and moderate levels of sexual IPV. 5.9% of our sample is classified in the “systematic IPV” subgroup and were exposed to both high levels of physical and sexual IPV in the past 12 months.

### Heterogeneity of program impacts by subgroups of physical and sexual IPV at baseline

We first explore the heterogenous impacts of EMAP by subgroups of couples defined using binary indicators of women’s experience of physical and sexual IPV at baseline. Table 4 reports the results among subgroups of physical IPV. While we do not detect any statistically significant impact of EMAP on the onset or cessation of physical or sexual IPV, we find that the program led to a statistically significant decrease (at the 10% level) in the severity indices of physical and sexual IPV at endline (physical IPV: β = −0.20; p<0.1; sexual IPV: β = −0.22; p<0.1) among couples exposed to physical IPV at baseline. The results also suggest that EMAP led to an increase in the severity of physical IPV among the couples not exposed to physical IPV at baseline (β = 0.12; p<0.1).

**Table 4:** Effects of EMAP on experience of IPV by subgroups of physical IPV experience at baseline.

		Mean of outcome (endline)	Treatment effect on subgroup (β)	Lower bound	Upper bound	p-value	N
**1. Outcome: Binary Physical IPV**							**943**
Subgroup: Physically Violent at baseline	EMAP	0.525	-0.100	-0.238	0.039	0.151	
	Control	0.659					
Subgroup: Not Physically violent at baseline	EMAP	0.265	0.017	-0.051	0.084	0.618	
	Control	0.242					
**2. Outcome: Physical IPV Severity Index**							**1003**
Subgroup: Physically Violent at baseline	EMAP	0.273	-0.202	-0.437	0.033	0.089	
	Control	0.443					
Subgroup: Not Physically violent at baseline	EMAP	-0.120	0.121	-0.001	0.243	0.052	
	Control	-0.222					
**3. Outcome: Binary Sexual IPV**							**948**
Subgroup: Physically Violent at baseline	EMAP	0.471	-0.052	-0.167	0.063	0.361	
	Control	0.545					
Subgroup: Not Physically Violent at baseline	EMAP	0.244	0.004	-0.091	0.098	0.933	
	Control	0.240					
**4. Outcome: Sexual IPV Severity Index**							**994**
Subgroup: Physically Violent at baseline	EMAP	0.285	-0.222	-0.477	0.033	0.085	
	Control	0.495					
Subgroup: Not Physically Violent at baseline	EMAP	-0.203	-0.019	-0.187	0.149	0.820	
	Control	-0.192					

Notes: OLS analysis. Adjusted for site pairs and baseline characteristics including household size, men and women’s age and education, and the language of the interview. Standards errors clustered at the site level. IPV severity indices are standardized for the ease of comparability. Number of observations for each specification is given in the last column.

Table 5 shows the results among subgroups of sexual IPV at baseline. We do not find any significant heterogenous effects of EMAP among these subgroups, suggesting that EMAP was not effective at preventing or reducing physical or sexual IPV regardless of the baseline sexual IPV status of the couple. These findings are robust to using alternative measures of severity indices of both physical and sexual IPV (See S1 Table 3 and 4 in the S1 Appendix).

**Table 5:** Effects of EMAP on experience of IPV by subgroups of sexual IPV experience at baseline.

		Mean of outcome (endline)	Treatment effect on subgroup (β)	Lower bound	Upper bound	p-value	N
**1. Outcome: Binary Physical IPV**							**962**
Subgroup: Sexually Violent at baseline	EMAP	0.474	-0.071	-0.170	0.028	0.152	
	Control	0.564					
Subgroup: Not Sexually violent at baseline	EMAP	0.299	0.005	-0.056	0.065	0.870	
	Control	0.291					
**2. Outcome: Physical IPV Severity Index**							**1024**
Subgroup: Sexually Violent at baseline	EMAP	0.288	0.003	-0.257	0.262	0.984	
	Control	0.270					
Subgroup: Not Sexually violent at baseline	EMAP	-0.090	0.034	-0.096	0.164	0.595	
	Control	-0.128					
**3. Outcome: Binary Sexual IPV**							**968**
Subgroup: Sexually Violent at baseline	EMAP	0.547	-0.004	-0.098	0.091	0.938	
	Control	0.575					
Subgroup: Not Sexually violent at baseline	EMAP	0.547	-0.006	-0.069	0.056	0.843	
	Control	0.575					
**4. Outcome: Sexual IPV Severity Index**							**1014**
Subgroup: Sexually Violent at baseline	EMAP	0.374	-0.095	-0.333	0.143	0.421	
	Control	0.462					
Subgroup: Not Sexually violent at baseline	EMAP	0.374	-0.052	-0.176	0.071	0.392	
	Control	0.462					

Notes: OLS analysis. Adjusted for site pairs and baseline characteristics including household size, men and women’s age and education, and the language of the interview. Standards errors clustered at the site level. IPV severity indices are standardized for the ease of comparability. Number of observations for each specification is given in the last column.

### Heterogeneity of program impacts by latent subgroups of couples at baseline

Turning to the analysis of the heterogenous impacts of EMAP by latent subgroups, we find statistically significant impacts of EMAP on both cessation and reduction of physical IPV exclusively among couples who were exposed to high physical and moderate sexual IPV at baseline. Table 6 shows that for this subgroup, EMAP decreases the probability of experiencing physical IPV at endline by 19.6 percentage points (p<0.05) and decreases the severity of physical IPV at endline by 0.64 standard deviations (p<0.05). These findings are robust to using alternative measures of severity of both physical and sexual IPV (See S1 Table 5 in the S1 Appendix).

**Table 6:** Effects of EMAP on experience of IPV by latent subgroups at baseline.

		Mean of outcome (endline)	Treatment effect on subgroup (β)	Lower bound	Upper bound	p-value	N
**1. Outcome: Binary Physical IPV**							**996**
Subgroup: Systematic IPV at baseline	EMAP	0.654	-0.031	-0.263	0.200	0.783	
	Control	0.735					
Subgroup: High Physical & Moderate Sexual IPV at baseline	EMAP	0.566	-0.196	-0.387	-0.006	0.044	
	Control	0.806					
Subgroup: Moderate Physical & High Sexual IPV at baseline	EMAP	0.461	-0.101	-0.241	0.039	0.149	
	Control	0.591					
Subgroup: Little to no IPV at baseline	EMAP	0.281	0.020	-0.037	0.077	0.482	
	Control	0.254					
**2. Outcome: Physical IPV Severity Index**							**1066**
Subgroup: Systematic IPV at baseline	EMAP	0.572	-0.337	-0.869	0.195	0.205	
	Control	0.834					
Subgroup: High Physical & Moderate Sexual IPV at baseline	EMAP	0.300	-0.643	-1.182	-0.103	0.021	
	Control	0.853					
Subgroup: Moderate Physical & High Sexual IPV at baseline	EMAP	0.225	0.108	-0.120	0.336	0.339	
	Control	0.155					
Subgroup: Little to no IPV at baseline	EMAP	-0.112	0.094	-0.020	0.208	0.101	
	Control	-0.203					
**3. Outcome: Binary Sexual IPV**							**998**
Subgroup: Systematic IPV at baseline	EMAP	0.538	-0.117	-0.369	0.136	0.352	
	Control	0.686					
Subgroup: High Physical & Moderate Sexual IPV at baseline	EMAP	0.472	-0.136	-0.355	0.084	0.215	
	Control	0.641					
Subgroup: Moderate Physical & High Sexual IPV at baseline	EMAP	0.622	0.042	-0.131	0.215	0.625	
	Control	0.576					
Subgroup: Little to no IPV at baseline	EMAP	0.225	0.001	-0.073	0.075	0.975	
	Control	0.229					
**4. Outcome: Sexual IPV Severity Index**							**1055**
Subgroup: Systematic IPV at baseline	EMAP	0.654	-0.026	-0.946	0.894	0.954	
	Control	0.708					
Subgroup: High Physical & Moderate Sexual IPV at baseline	EMAP	0.347	-0.461	-1.016	0.094	0.100	
	Control	0.831					
Subgroup: Moderate Physical & High Sexual IPV at baseline	EMAP	0.421	-0.155	-0.595	0.284	0.475	
	Control	0.509					
Subgroup: Little to no IPV at baseline	EMAP	-0.235	-0.019	-0.159	0.122	0.788	
	Control	-0.216					

Notes: OLS analysis. Adjusted for site pairs and baseline characteristics including household size, men and women’s age and education, and the language of the interview. Standards errors clustered at the site level. IPV severity indices are standardized for the ease of comparability. Number of observations for each specification is given in the last column.

We also find a non-statistically significant decrease in the severity of sexual IPV among couples exposed to high physical and moderate sexual IPV at baseline (β = −0.46; p = 0.1). When using alternative measures of severity of sexual IPV, we see a statistically significant reduction at the 5% significance level. This latter finding contrasts with findings from the analysis by subgroups using binary indicators of experience of IPV at baseline.

## Conclusions

The overarching objective of the EMAP program is to promote gender equality and reduce IPV in communities through building a cadre of male allies. The program was originally intended to include only non-violent men and to build their skills for championing gender equality and non-violence. However, given difficulties in screening for IPV perpetration as part of program recruitment criteria, particularly in settings with highly endemic violence such as eastern DRC, the program included a non-negligeable proportion of men who had used violence against their intimate partner. It is therefore critical to examine whether the program has differential impacts on men who were more or less violent at the start.

In this paper, we show that the EMAP program was effective at reducing and stopping physical IPV among men who were most physically violent in the year before the start of the program, although these men were not the target population of the program. LCA identifies four distinct groups of couples based on female partners’ reports of experience of violence at baseline. The largest class include couples exposed to little to no violence in the 12 months preceding the baseline survey (70% of participants). Couples exposed to high levels of sexual and/or physical IPV were in the minority. This program composition may explain some of the results highlighted in this paper.

This study has a number of limitations. Firstly, several of the subgroups obtained through the LCA are small, leading to a loss in statistical power to detect differences between groups. In addition, and as already noted in Vaillant et al. [5], study outcomes are self-reported and may be subject to social desirability bias that would lead to under-reporting or over-reporting. Using self-administrated questionnaires for sensitive questions was intended to mitigate this risk. Finally, our findings rely on a short-term follow-up: outcomes were collected around 9.5 months after the end of the intervention. We cannot exclude that the observed heterogenous effects of the programe were different in the long run.

Research on the effects of social norms at the individual level focuses on the influence of both descriptive social norms (defined as the perceived prevalence of a behavior), and injunctive social norms (defined as the acceptability of the behavior) [25]. Evidence from the baseline survey and an accompanying qualitative study indicate that high levels of violence are not valorized among program participants. At baseline, fewer than ten percent of men agreed that a man is justified in beating his wife if she goes out without telling him, neglects the children, argues with him, refuses to have sex, or burns the food. The qualitative research found that physical violence against women was tolerated as a means of enforcing male superiority in the household, but violence was not celebrated [16]. Thus, high levels of violence were neither common nor admired by the majority of EMAP participants.

While we do not have a direct measure of perceived norms, the baseline measures of behaviors and attitudes suggest that EMAP participants regarded only low levels of IPV as common and acceptable. The results presented in this paper suggest that men who were using higher than average levels of violence were encouraged by the program to reduce the severity of the violence that they perpetrate. In contrast, there was no impact of the program on the prevention, cessation, or reduction of physical or sexual violence among couples exposed to little or no IPV in the year before baseline. On the contrary, the analysis suggests that the program may have had unintended negative effects on couples who were not exposed to physical IPV before the start of the intervention, as the program led to increases in severity of physical violence among those couples. In sum, EMAP seems to have affected the behavior of men who had used higher than average levels of violence in the year before baseline but did not positively affect the behavior of men whose actions seemed to be in line with existing group norms.

These results suggest that social learning took place within the EMAP groups. Men whose baseline use of violence was above average may have learned that their behavior was inconsistent with the preferences or actions of others in their EMAP group. Alternatively, the group discussions may have caused shifts in their expectations about the likelihood or gravity of sanctions for their non-normative behavior. These same processes could also explain the slight increase in the use of violence among men who were perpetrating little to no violence at baseline if those men learned that some levels of violence are unlikely to lead to social sanctions. These mechanisms of impact should be investigated in future studies. If these hypothesized mechanisms do, in fact, play a role in promoting change, then the composition of discussion groups will be a critical feature of program design. Groups that are comprised of both more and less violent men may be important for encouraging progress among “outlier” men using violence levels above the group norm. Furthermore, as noted by Gibbs et al. [4], the normative context will influence whose behavior is most likely to change in response to prevention programming. Given that it is not ethically responsible or feasible to screen for IPV perpetration as part of program recruitment, implementers should be attentive to prevailing norms and rates of IPV perpetration as important contextual factors that will likely influence group dynamics.

The findings have two important implications for future research. First, studies focusing on measuring only cessation of IPV are likely to miss important changes in the severity of violence. Estimating reduction in severity of IPV should be systematically performed when there is reason to believe that a program cannot realistically achieve a normative shift to a new consensus of zero tolerance for violence within the study timeframe. Second, given the possibility for heterogeneous impacts of interventions such as the EMAP that rely on group interactions, it is important for researchers to plan for such analyses at the design phase. Limited sample size and related lack of statistical power has been a recurrent issue in the studies of violence prevention. This means that sample sizes should be sufficiently large to detect changes in subgroups, rather than only average changes in the treatment group.

It is also important to note that the program was not as successful at addressing sexual IPV than physical IPV, despite both types of violence being addressed in the program content. There are several possible explanations for the mixed effects on levels of sexual violence. First, because sexual violence often occurs in private and is not observable, the behavior may be more weakly influenced by the potential for social sanctions [26]. Second, existing research suggests that motivations for physical and sexual violence are different, with physical violence being used more instrumentally to extract resources and sexual violence being driven by men’s expressive desires [27]. Different strategies may be necessary to curb these different types of violence. Finally, other research in Central/East Africa finds that it is especially difficult to change rates of sexual violence within marriage because prevailing norms indicate that men have unrestricted rights to sex with their wives [28]. Future programming and research should be attentive to specific conditions and requirements for reducing sexual violence.

This analysis of the heterogeneous impacts of the EMAP program shows that in contexts of endemic violence, programs like EMAP can lead to a meaningful short-term reduction in harm to women, perhaps even without transforming prevailing norms about male superiority or the acceptability of IPV. While a fundamental shift in gender relations is an essential goal for the promotion of gender equality and women’s rights, it is important to measure shorter-term improvements as well. Careful analysis of incremental changes can also be useful for learning about how best to promote systemic transformative change in the future.

## Supporting information

S1 Fig Trial profile. (TIF)

S1 Appendix (DOCX)

S1 Dataset (DTA)
