# Supplementary figures and images for: Prevention, Cessation, or harm reduction: Heterogeneous effects of an intimate partner violence prevention program in eastern Democratic Republic of the Congo
Source: PLoS One. 2023 Mar 8;18(3):e0282339. doi: 10.1371/journal.pone.0282339 (PMC9994709; doi:10.1371/journal.pone.0282339)

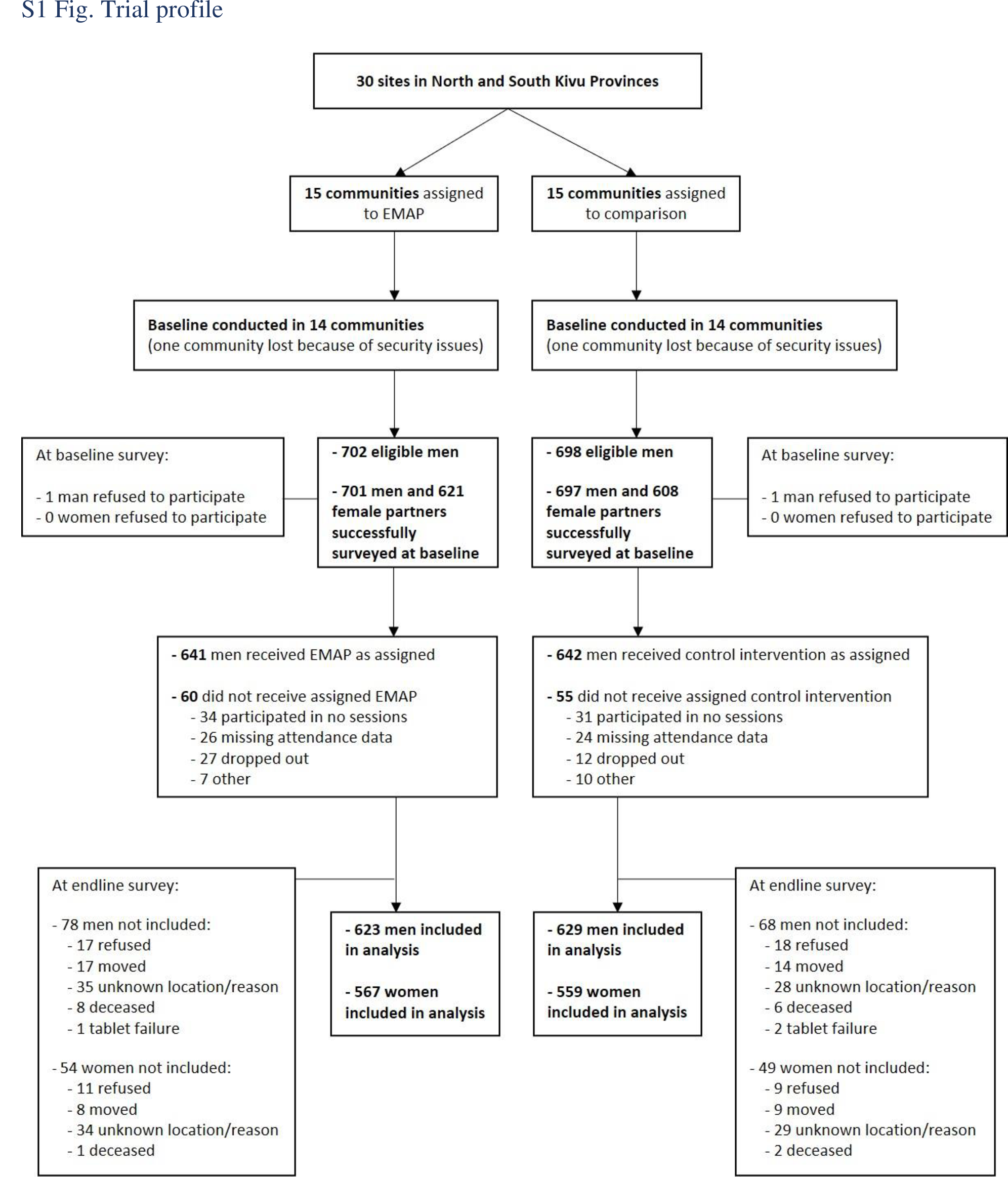

Supplement: S1 Fig — (TIF) [file pone.0282339.s001.tif]
