# Supplementary material for: Prevention, Cessation, or harm reduction: Heterogeneous effects of an intimate partner violence prevention program in eastern Democratic Republic of the Congo
Source: PLoS One. 2023 Mar 8;18(3):e0282339. doi: 10.1371/journal.pone.0282339 (PMC9994709; doi:10.1371/journal.pone.0282339)
Supplement: S1 Appendix — (DOCX) [file pone.0282339.s002.docx]

## S1 Appendix

### Table A1. Physical and sexual IPV acts at baseline

|  | **N** | **Mean** |
| --- | --- | --- |
| ***Physical IPV acts*** |  |  |
| Partner pushed, shaken, or thrown something at respondent | 1183 | 0.199 |
| Partner slapped respondent | 1175 | 0.203 |
| Partner twisted respondent's arm or pulled her hair | 1178 | 0.113 |
| Partner punched respondent with his fist or something that could hurt her | 1173 | 0.118 |
| Partner kicked respondent, dragged her on the floor, beat her up | 1178 | 0.114 |
| Partner tried to choke respondent or burn her on purpose | 1185 | 0.058 |
| Partner threatened or attacked her with a knife, gun, or other weapon | 1183 | 0.049 |
| ***Sexual IPV acts*** |  |  |
| Partner physically forced respondent to have sex with him even when she did not want to | 1189 | 0.234 |
| Partner forced respondent to have sex even when she did not want to because she was afraid of what he could do to her | 1183 | 0.163 |
| Partner forced respondent to do sexual acts respondent finds humiliating | 1178 | 0.141 |

### Table A2. Model fit for latent class models

| **Number of Classes** | **AIC** | **BIC** | **Log-likelihood** | **Entropy** |
| --- | --- | --- | --- | --- |
| 2 | 7368.563 | 7475.802 | -3663.281 | 0.88 |
| 3 | 7123.029 | 7286.44 | -3529.514 | 0.84 |
| 4 | 7058.022 | 7277.606 | -3486.011 | 0.85 |

### Table A3. Effects of EMAP on experience of IPV by subgroups of physical IPV experience at baseline: robustness checks using alternative constructions of indices

|  |  | Mean of outcome (endline) | Treatment effect on subgroup (β) | Lower bound | Upper bound | p-value | N |  |
| --- | --- | --- | --- | --- | --- | --- | --- | --- |
| **1.Outcome: Physical IPV Severity Index^a^** |  |  |  |  |  |  | **864** |  |
| Subgroup: Physically Violent at baseline | EMAP | 0.268 | -0.293 | -0.579 | -0.006 | 0.046 |  |  |
|  | Control | 0.541 |  |  |  |  |  |  |
| Subgroup: Not Physically violent at baseline | EMAP | -0.162 | 0.010 | -0.108 | 0.128 | 0.861 |  |  |
|  | Control | -0.168 |  |  |  |  |  |  |
| **2.Outcome: Physical IPV Severity Index^b^** |  |  |  |  |  |  | **1003** |  |
| Subgroup: Physically Violent at baseline | EMAP | 0.275 | -0.211 | -0.465 | 0.042 | 0.098 |  |  |
|  | Control | 0.471 |  |  |  |  |  |  |
| Subgroup: Not Physically violent at baseline | EMAP | -0.142 | 0.077 | -0.038 | 0.192 | 0.181 |  |  |
|  | Control | -0.207 |  |  |  |  |  |  |
| **3.Outcome: Physical IPV Severity Index^c^** |  |  |  |  |  |  | **1003** |  |
| Subgroup: Physically Violent at baseline | EMAP | 0.283 | -0.216 | -0.468 | 0.035 | 0.089 |  |  |
|  | Control | 0.480 |  |  |  |  |  |  |
| Subgroup: Not Physically violent at baseline | EMAP | -0.145 | 0.086 | -0.032 | 0.203 | 0.145 |  |  |
|  | Control | -0.218 |  |  |  |  |  |  |
| **4.Outcome: Sexual IPV Severity Index^a^** |  |  |  |  |  |  | **913** |  |
| Subgroup: Physically Violent at baseline | EMAP | 0.252 | -0.214 | -0.469 | 0.041 | 0.097 |  |  |
|  | Control | 0.484 |  |  |  |  |  |  |
| Subgroup: Not Physically violent at baseline | EMAP | -0.184 | -0.039 | -0.214 | 0.137 | 0.655 |  |  |
|  | Control | -0.168 |  |  |  |  |  |  |
| **5.Outcome: Sexual IPV Severity Index^b^** |  |  |  |  |  |  | **994** |  |
| Subgroup: Physically Violent at baseline | EMAP | 0.259 | -0.238 | -0.472 | -0.004 | 0.046 |  |  |
|  | Control | 0.497 |  |  |  |  |  |  |
| Subgroup: Not Physically violent at baseline | EMAP | -0.196 | -0.028 | -0.197 | 0.142 | 0.740 |  |  |
|  | Control | -0.181 |  |  |  |  |  |  |
| **6.Outcome: Sexual IPV Severity Index^c^** |  |  |  |  |  |  | **994** |  |
| Subgroup: Physically Violent at baseline | EMAP | 0.261 | -0.238 | -0.476 | 0.000 | 0.050 |  |  |
|  | Control | 0.499 |  |  |  |  |  |  |
| Subgroup: Not Physically violent at baseline | EMAP | -0.199 | -0.025 | -0.197 | 0.147 | 0.769 |  |  |
|  | Control | -0.184 |  |  |  |  |  |  |
| Notes: OLS analysis. Adjusted for site pairs and baseline characteristics including household size, men and women’s age, and education, and the language of the interview. Standards errors clustered at the site level. IPV indices are standardized for the ease of comparability. a: The index is the sum of the item scores where it is coded to missing if any item was missing. b: The index is the sum of item scores where the missing items are replaced with “0=Never” if there is at least one non-missing item. c: The index is the sum of the item scores where the missing items are replaced with the mean of the item in the control group if there is at least one non-missing item. Number of observations for each specification is given in the last column. | | | | | | | | |

### Table A4. Effects of EMAP on experience of IPV by subgroups of sexual IPV experience at baseline: robustness checks using alternative constructions of severity indices

|  |  | Mean of outcome (endline) | Treatment effect on subgroup (β) | Lower bound | Upper bound | p-value | N |
| --- | --- | --- | --- | --- | --- | --- | --- |
| **1.Outcome: Physical IPV Severity Index^a^** |  |  |  |  |  |  | **881** |
| Subgroup: Sexually Violent at baseline | EMAP | 0.147 | -0.098 | -0.348 | 0.153 | 0.432 |  |
|  | Control | 0.275 |  |  |  |  |  |
| Subgroup: Not Sexually violent at baseline | EMAP | 0.147 | -0.064 | -0.199 | 0.071 | 0.339 |  |
|  | Control | 0.275 |  |  |  |  |  |
| **2.Outcome: Physical IPV Severity Index^b^** |  |  |  |  |  |  | **1024** |
| Subgroup: Sexually Violent at baseline | EMAP | 0.191 | -0.005 | -0.245 | 0.234 | 0.964 |  |
|  | Control | 0.247 |  |  |  |  |  |
| Subgroup: Not Sexually violent at baseline | EMAP | 0.191 | -0.008 | -0.136 | 0.121 | 0.906 |  |
|  | Control | 0.247 |  |  |  |  |  |
| **3.Outcome: Physical IPV Severity Index^c^** |  |  |  |  |  |  | **1024** |
| Subgroup: Sexually Violent at baseline | EMAP | 0.202 | -0.008 | -0.253 | 0.237 | 0.945 |  |
|  | Control | 0.254 |  |  |  |  |  |
| Subgroup: Not Sexually violent at baseline | EMAP | 0.202 | 0.001 | -0.128 | 0.129 | 0.991 |  |
|  | Control | 0.254 |  |  |  |  |  |
| **4.Outcome: Sexual IPV Severity Index^a^** |  |  |  |  |  |  | **935** |
| Subgroup: Sexually Violent at baseline | EMAP | 0.358 | -0.123 | -0.343 | 0.098 | 0.263 |  |
|  | Control | 0.508 |  |  |  |  |  |
| Subgroup: Not Sexually violent at baseline | EMAP | 0.358 | -0.042 | -0.173 | 0.088 | 0.511 |  |
|  | Control | 0.508 |  |  |  |  |  |
| **5.Outcome: Sexual IPV Severity Index^b^** |  |  |  |  |  |  | **1014** |
| Subgroup: Sexually Violent at baseline | EMAP | 0.334 | -0.133 | -0.345 | 0.079 | 0.210 |  |
|  | Control | 0.470 |  |  |  |  |  |
| Subgroup: Not Sexually violent at baseline | EMAP | 0.334 | -0.051 | -0.179 | 0.077 | 0.420 |  |
|  | Control | 0.470 |  |  |  |  |  |
| **6.Outcome: Sexual IPV Severity Index^c^** |  |  |  |  |  |  | **1014** |
| Subgroup: Sexually Violent at baseline | EMAP | 0.350 | -0.124 | -0.339 | 0.092 | 0.249 |  |
|  | Control | 0.481 |  |  |  |  |  |
| Subgroup: Not Sexually violent at baseline | EMAP | 0.350 | -0.052 | -0.180 | 0.076 | 0.416 |  |
|  | Control | 0.481 |  |  |  |  |  |
| Notes: OLS analysis. Adjusted for site pairs and baseline characteristics including household size, men and women’s age, and education, and the language of the interview. Standards errors clustered at the site level. IPV indices are standardized for the ease of comparability. a: The index is the sum of the item scores where it is coded to missing if any item was missing. B: The index is the sum of item scores where the missing items are replaced with “0=Never” if there is at least one non-missing item. c: The index is the sum of the item scores where the missing items are replaced with the mean of the item in the control group if there is at least one non-missing item. Number of observations for each specification is given in the last column. | | | | | | | |

### Table A5. Effects of EMAP on experience of IPV by latent subgroups: robustness checks using alternative constructions of severity indices

|  |  | Mean of outcome (endline) | Treatment effect on subgroup (β) | Lower bound | Upper bound | p-value | N |  |
| --- | --- | --- | --- | --- | --- | --- | --- | --- |
| **1.Outcome: Physical IPV Severity Index^a^** |  |  |  |  |  |  | **909** |  |
| Subgroup: Systematic IPV at baseline | EMAP | 0.913 | -0.058 | -0.924 | 0.808 | 0.891 |  |  |
|  | Control | 0.927 |  |  |  |  |  |  |
| Subgroup: High Physical & Moderate Sexual IPV at baseline | EMAP | 0.170 | -0.960 | -1.686 | -0.233 | 0.012 |  |  |
|  | Control | 1.114 |  |  |  |  |  |  |
| Subgroup: Moderate Physical & High Sexual IPV at baseline | EMAP | 0.183 | 0.040 | -0.202 | 0.282 | 0.738 |  |  |
|  | Control | 0.168 |  |  |  |  |  |  |
| Subgroup: Little to no IPV at baseline | EMAP | -0.154 | -0.017 | -0.139 | 0.105 | 0.780 |  |  |
|  | Control | -0.143 |  |  |  |  |  |  |
| **2.Outcome: Physical IPV Severity Index^b^** |  |  |  |  |  |  | **1066** |  |
| Subgroup: Systematic IPV at baseline | EMAP | 0.647 | -0.262 | -0.835 | 0.312 | 0.358 |  |  |
|  | Control | 0.849 |  |  |  |  |  |  |
| Subgroup: High Physical & Moderate Sexual IPV at baseline | EMAP | 0.247 | -0.734 | -1.335 | -0.133 | 0.019 |  |  |
|  | Control | 0.948 |  |  |  |  |  |  |
| Subgroup: Moderate Physical & High Sexual IPV at baseline | EMAP | 0.201 | 0.099 | -0.104 | 0.301 | 0.326 |  |  |
|  | Control | 0.140 |  |  |  |  |  |  |
| Subgroup: Little to no IPV at baseline | EMAP | -0.131 | 0.056 | -0.060 | 0.173 | 0.329 |  |  |
|  | Control | -0.185 |  |  |  |  |  |  |
| **3.Outcome: Physical IPV Severity Index^c^** |  |  |  |  |  |  | **1066** |  |
| Subgroup: Systematic IPV at baseline | EMAP | 0.672 | -0.256 | -0.830 | 0.319 | 0.369 |  |  |
|  | Control | 0.860 |  |  |  |  |  |  |
| Subgroup: High Physical & Moderate Sexual IPV at baseline | EMAP | 0.258 | -0.735 | -1.325 | -0.145 | 0.016 |  |  |
|  | Control | 0.954 |  |  |  |  |  |  |
| Subgroup: Moderate Physical & High Sexual IPV at baseline | EMAP | 0.202 | 0.086 | -0.121 | 0.293 | 0.399 |  |  |
|  | Control | 0.153 |  |  |  |  |  |  |
| Subgroup: Little to no IPV at baseline | EMAP | -0.130 | 0.067 | -0.049 | 0.183 | 0.249 |  |  |
|  | Control | -0.193 |  |  |  |  |  |  |
| **4.Outcome: Sexual IPV Severity Index^a^** |  |  |  |  |  |  | **961** |  |
| Subgroup: Systematic IPV at baseline | EMAP | 0.690 | -0.125 | -0.985 | 0.734 | 0.767 |  |  |
|  | Control | 0.820 |  |  |  |  |  |  |
| Subgroup: High Physical & Moderate Sexual IPV at baseline | EMAP | 0.333 | -0.344 | -0.832 | 0.143 | 0.158 |  |  |
|  | Control | 0.709 |  |  |  |  |  |  |
| Subgroup: Moderate Physical & High Sexual IPV at baseline | EMAP | 0.369 | -0.101 | -0.482 | 0.279 | 0.590 |  |  |
|  | Control | 0.466 |  |  |  |  |  |  |
| Subgroup: Little to no IPV at baseline | EMAP | -0.217 | -0.046 | -0.200 | 0.108 | 0.545 |  |  |
|  | Control | -0.183 |  |  |  |  |  |  |
| **5.Outcome: Sexual IPV Severity Index^b^** |  |  |  |  |  |  | **1055** |  |
| Subgroup: Systematic IPV at baseline | EMAP | 0.542 | -0.202 | -1.016 | 0.611 | 0.614 |  |  |
|  | Control | 0.773 |  |  |  |  |  |  |
| Subgroup: High Physical & Moderate Sexual IPV at baseline | EMAP | 0.327 | -0.444 | -0.921 | 0.033 | 0.067 |  |  |
|  | Control | 0.798 |  |  |  |  |  |  |
| Subgroup: Moderate Physical & High Sexual IPV at baseline | EMAP | 0.385 | -0.142 | -0.535 | 0.251 | 0.464 |  |  |
|  | Control | 0.476 |  |  |  |  |  |  |
| Subgroup: Little to no IPV at baseline | EMAP | -0.228 | -0.029 | -0.176 | 0.117 | 0.687 |  |  |
|  | Control | -0.201 |  |  |  |  |  |  |
| **6.Outcome: Sexual IPV Severity Index^c^** |  |  |  |  |  |  | **1055** |  |
| Subgroup: Systematic IPV at baseline | EMAP | 0.584 | -0.159 | -0.977 | 0.660 | 0.693 |  |  |
|  | Control | 0.764 |  |  |  |  |  |  |
| Subgroup: High Physical & Moderate Sexual IPV at baseline | EMAP | 0.329 | -0.444 | -0.930 | 0.041 | 0.071 |  |  |
|  | Control | 0.802 |  |  |  |  |  |  |
| Subgroup: Moderate Physical & High Sexual IPV at baseline | EMAP | 0.397 | -0.144 | -0.534 | 0.246 | 0.455 |  |  |
|  | Control | 0.490 |  |  |  |  |  |  |
| Subgroup: Little to no IPV at baseline | EMAP | -0.233 | -0.029 | -0.177 | 0.119 | 0.687 |  |  |
|  | Control | -0.204 |  |  |  |  |  |  |
| Notes: OLS analysis. Adjusted for site pairs and baseline characteristics including household size, men and women’s age, and education, and the language of the interview. Standards errors clustered at the site level. IPV indices are standardized for the ease of comparability. a: The index is the sum of the item scores where it is coded to missing if any item was missing. b: The index is the sum of item scores where the missing items are replaced with “0=Never” if there is at least one non-missing item. c: The index is the sum of the item scores where the missing items are replaced with the mean of the item in the control group if there is at least one non-missing item. Number of observations for each specification is given in the last column. | | | | | | | | |
